# Supplementary material for: The Activation of PPARγ by (2Z,4E,6E)-2-methoxyocta-2,4,6-trienoic Acid Counteracts the Epithelial–Mesenchymal Transition Process in Skin Carcinogenesis
Source: Cells. 2023 Mar 24;12(7):1007. doi: 10.3390/cells12071007 (PMC10093137; doi:10.3390/cells12071007)
Supplement: Supplementary file 1 [file cells-12-01007-s001.zip › cells-2183609-supplementary.pdf]

Figure S1 Effects of Octa and A02 on A431 cells viability.

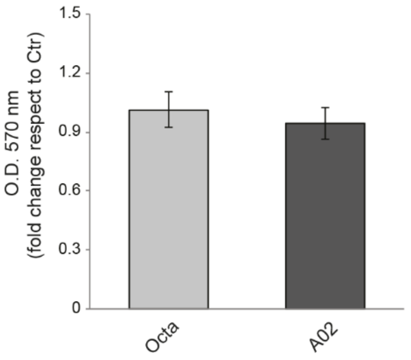

Cell viability evaluated by MTT assay on NHKs treated with Octa or A02 (90 µM) for 72 h. The data in the graphs are mean ± SD of three independent experiments.

Figure S2 Effects of A02 and Octa on the differentiation process in NHKs.

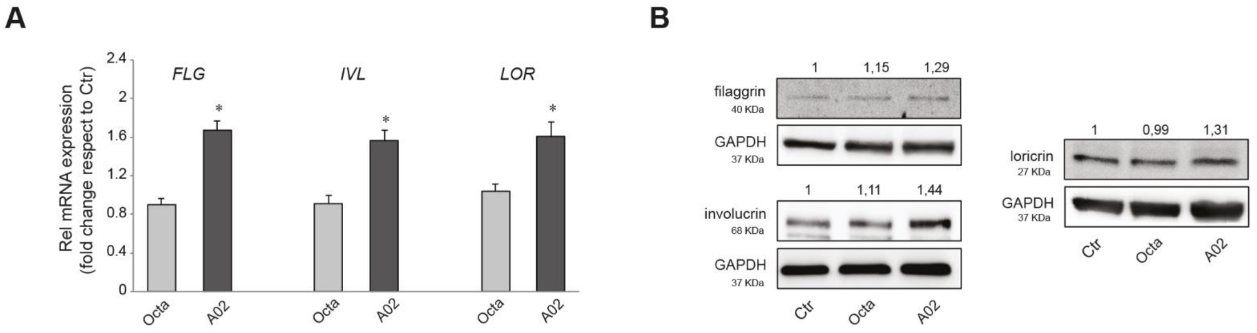

(A) Quantitative real-time PCR analysis of FLG, IVL, and LOR in NHKs incubated with Octa or A02 (90 µM) for 48 h. All mRNA values were normalized against the expression of GAPDH and were expressed relative to untreated control cells. The data in the graphs are mean ± SD of three independent experiments (*$p<0.05$ vs untreated control). (B) Western blot analysis of filaggrin, involucrin and loricrin protein expression in NHKs treated with A02 (90 µM) for 72 h. Representative blots are shown. GAPDH was used as an endogenous loading control for western blot analysis. Densitometric scanning of band intensities was performed to quantify the change in protein expression. Data represent the mean ± SD of three independent experiments and are expressed as fold change respect to untreated control cells (control value taken as 1-fold in each case).

Figure S3 Experimental design and histological analysis of DMBA-TPA-induced mouse skin papillomas.

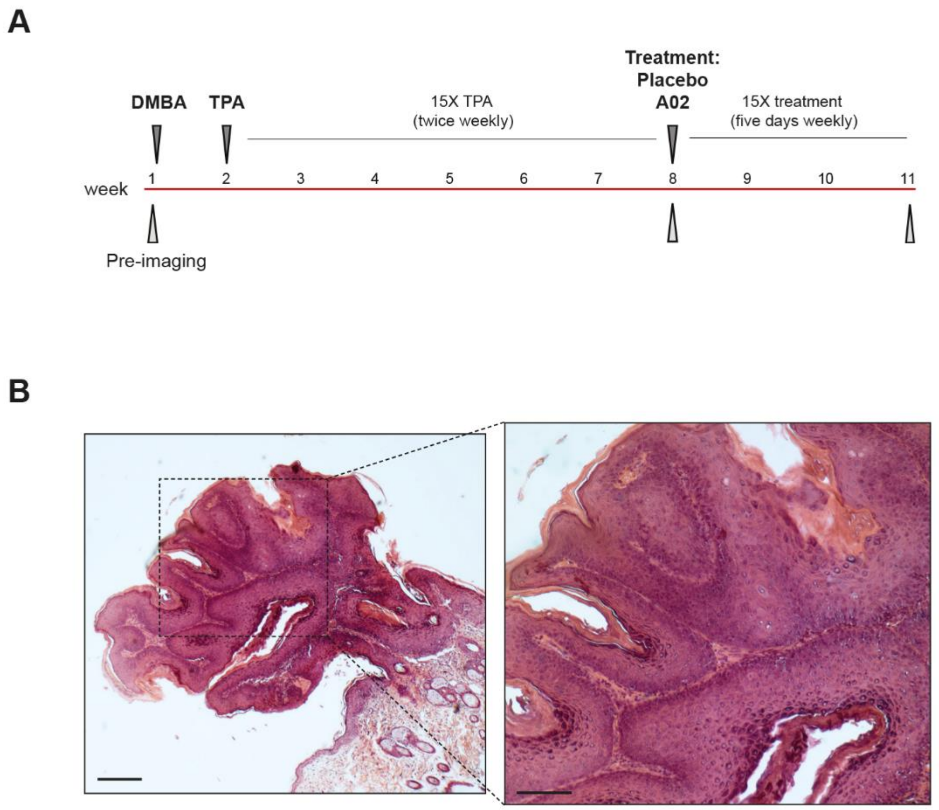

(A) Schematic representation of the experimental design. (B) Representative images of Hematoxylin & Eosin stained sections from mouse skin after 1X DMBA and 15X TPA. Histological features of skin papillomas are represented by acanthotic epidermis, cytoarchitectural anomalies of basal and suprabasal epithelium, dermal fibroblast proliferation and inflammatory cell infiltrate. The boxed area represents the enlarged view of the selected frame. Bars: 200 μm; 100 μm.

Table S1. Human primers used for the Real time RT-PCR analysis.

Gene	Oligonucleotide sequences (5'-3')	Amplicon size	Accession Number
Catalase	*For:* TTTCCCAGGAAGATCCTGAC<br>*Rev:* ACCTTGGTGAGATCGAATGG	148 bp	NM_001752.4
E-Cadherin	*For:* GAACGCATTGCCACATACAC<br>*Rev:* ATTCGGGCTTGTTGTCATTC	118 bp	NM_004360.5
Fibronectin	*For:* CCTCGAAGAGCAAGAGGCAG<br>*Rev:* GCTTCAGGTTTACTCTCGCA	202 bp	NM_001365522.2
Filaggrin	*For:* GAAGACAAGGATCGCACCAG<br>*Rev:* ATGGTGTCCTGACCCTCTTG	76 bp	NM_002016.2
GAPDH	*For:* TGCACCACCAACTGCTTAGC<br>*Rev:* GGCATGGACTGTGGTCATGAG	198 bp	NM_001289746
HO-1	*For:* CCAGCGGGCCAGCAACAAAGTGC<br>*Rev:* AGCCTTCAGTGCCCACGGTAAGG	265 bp	NM_002133.3
Involucrin	*For:* ACCCATCAGGAGCAAATGAAA<br>*Rev:* GCTCGACAGGCACCTTCTGGC	67 bp	NM_005547.4
Loricrin	*For:* TCATGATGCTACCCGAGGTTTG<br>*Rev:* CAGAACTAGATGCAGCCGGAGA	87 bp	NM_000427.3
MMP2	*For:* AGAAGGCTGTGTTCTTTGCAG<br>*Rev:* AGGCTGGTCAGTGGCTTG	88 bp	NM_004530.6
N-Cadherin	*For:* GGACTATGATTACCTGAACGACTG<br>*Rev:* AGTTAAAGCCTAGCTTCTGAATGC	161 bp	NM_001792.5
NQO1	*For:* GGATTGGACCGAGCTGGAA<br>*Rev:* ATTGCAGTGAAGATGAAGGCAAC	140 bp	NM_000903.2
PPARγ	*For:* GCCAAGCTGCTCCAGAAAAT<br>*Rev:* TGATCACCTGCAGTAGCTGCA	73 bp	NM_138711
SLUG	*For:* TGGTTGCTTCAAGGACACAT<br>*Rev:* GCAAATGCTCTGTTGCAGTG	77 bp	NM_003068.5
Vimentin	*For:* TCTACGAGGAGGAGATGCGG<br>*Rev:* GGTCAAGACGTGCCAGAGAC	213 bp	NM_003380.5

Table S2. Mouse primers used for the Real time RT-PCR analysis.

Gene	Oligonucleotide sequences (5'-3')	Amplicon size	Accession Number
E-Cadherin	*For:* AAGTGACCGATGATGATGCC<br>*Rev:* CTTCATTCACGTCTACCACGT	258 bp	NM_009864.3
Fibronectin	*For:* CGAGGTGACAGAGACCACAA<br>*Rev:* CTGGAGTCAAGCCAGACACA	149 bp	NM_010233.2
GAPDH	*For:* TGCACCACCAACTGCTTAGC<br>*Rev:* GGCATGGACTGTGGTCATGAG	198 bp	NM_001289746
MMP2	*For:* AGAAGGCTGTGTTCTTTGCAG<br>*Rev:* AGGCTGGTCAGTGGCTTG	88 bp	NM_004530.6
N-Cadherin	*For:* TGAAACGGCGGGATAAAGAG<br>*Rev:* GGCTCCACAGTATCTGGTTG	157 bp	NM_007664.5
α-SMA	*For:* GAGGCACCACTGAACCCTAA<br>*Rev:* CATCTCCAGAGTCCAGCACA	154 bp	NM_007392.3
SLUG	*For:* CATTGCCTTGTGTCTGCA<br>*Rev:* AGAAAGGCTTTTCCCCAGTG	96 bp	NM_011415.3
Vimentin	*For:* TCTACGAGGAGGAGATGCGG<br>*Rev:* GGTCAAGACGTGCCAGAGAC	213 bp	NM_003380.5
